# Avortements non médicalisés: état des lieux à travers une étude rétrospective de 451 cas traités à l'hôpital militaire d'instruction Moulay Ismail Meknès, Maroc

**DOI:** 10.11604/pamj.2016.24.83.8624

**Published:** 2016-05-25

**Authors:** Omar Laghzaoui

**Affiliations:** 1 Université Sidi Mohammed Ben Abdellah Faculté de Médecine et de Pharmacie, Fès, Maroc

**Keywords:** Avortement, non médicalisé, complication, contraception, Abortion, unsafe, complication, contraception

## Abstract

L'avortement non médicalisé est un fléau dans le monde entier mais notamment en Afrique où il constitue un marché lucratif pour les tradipraticiens qui exposent les patientes à des complications graves voir mortelles. Notre étude rétrospective de 451 dossiers représente un échantillon exhaustif de cette pratique au Maroc soulevant les raisons qui poussent les femmes à l'avortement non médicalisé, la tranche d’âge la plus concernée, les conditions et les complications inhérentes à l'acte. Notre travail aboutit sur la nécessité d'intervenir avant la grossesse en développant la notion de contraception chez les jeunes filles dès la scolarité pour minimiser ces pratiques non sanitaires.

## Abstract (en)

Unsafe abortion is a plague spreading around the world but especially in Africa where it is a lucrative market for traditional practitioners which exposes patients to serious complications including death. Our retrospective study of 451 cases represent an exhaustive sample of this practice in Morocco exploring the reasons why women experience an unsafe abortion, what age group is most affected, conditions and complications inherent in this practice. Our study focuses on the need for intervention before pregnancy by developing the concept of contraception among girls from their early years to minimize these non-health practices.

## Introduction

L'avortement non médicalisé existe encore dans les pays où cet acte a été légalisé et codifié mais sa fréquence tend à s’élever surtout en Afrique et principalement dans les pays qui interdisent l'avortement. Notre travail consiste à réaliser un inventaire épidémiologique et clinique de ces pratiques à travers une étude rétrospective de 451 cas d'avortement non médicalisé pour relever les sites d'intervention afin d'atténuer au mieux les complications qui en résulte.

## Méthodes

C'est une étude rétrospective étalée sur une période de cinq ans du premier janvier 2009 au 30 décembre 2014 dénombrant 451 dossiers de patientes ayant étés victimes d'avortement non médicalisés sur 12040 grossesses prises en charge au service de gynécologie-obstétrique, Hôpital militaire Moulay Ismail Meknès. L’étude des dossiers a permis le recueil du profil épidémiologique, le statut de vaccination, les raisons ayant poussés les patientes à cette pratique, les circonstances de réalisation de l'acte, le motif clinique de l'admission à l'hôpital, les complications encourues et les moyens de prise en charge.

## Résultats

**Le profil épidémiologique:** La fréquence de l'avortement non médicalisé est de 3,74%, l’âge moyen des patientes est de 17 ans avec 70% entre 16 et 18 ans et des extrêmes allant de 15 à 42 ans. Niveau d'instruction des patientes: non instruites 62%, niveau primaire 31,9%, niveau secondaire 6,1%. 74,9% des femmes sont des primigestes non mariée avec grossesse illégitime ne désirant pas la grossesse par peur du jugement social et la perte de scolarité. 25,1% des patientes mariées avec grossesse non désirée en raison de situation financière incommode ou la vie du couple n'est pas vraiment stable (les maris sont souvent en déplacement pour missions militaires de longue durée).

**Le statut de vaccination:** 93,4% des patientes sont vaccinées: BCG, diphtérie, tétanos, coqueluche poliomyélite, la rougeole, les oreillons et la rubéole, 20% sont à jour pour le rappel du vaccin antitétanique.

**Le motif d'admission:** 85,1% des patientes sont recrutées par le billet des urgences, 14,9% ont consultées dans des formations privées puis diriger directement au service de gynécologie. Les motifs conduisant à la consultation dans notre formation hospitalière sont représentés sur le Tableau 1.

**Tableau 1:** Les motifs de consultation des patientes victimes d'avortements non médicalisés

Motif de consultation	Nombre de patientes	Pourcentage
Hémorragie génitale	252	55,7%
Hyperthermie	171	37,8%
Algie pelvienne	156	34,5%
Leucorrhées	150	33,2%
Syncope	11	2,4%

**Les lieux et la qualification des prestataires de l'avortement** La majeure partie des avortements se sont passés à domicile (295 patientes soit 65,41%), pratiqués par des accoucheuses traditionnelles dans des conditions déplorables. 106 patientes (23,51%) ont avorté dans des infirmeries de quartier ou des cabinets privés par du personnel non qualifié pour cet acte (infirmiers, médecins généralistes). 50 patientes (11,08%) ont elles-mêmes utilisé des procédés pour l'avortement.

**Les moyens utilisés pour l'avortement:** Plusieurs moyens sont utilisés pour l'avortement avec des fois l'association de plusieurs substances. Le Tableau 2 englobe les différents moyens qui ont servis pour l'avortement.

**Tableau 2:** Les moyens abortifs utilisés

Voie d'utilisation	Les moyens abortifs
Orale	- Infusions: la cannelle, le crocus, le persil, et l'absinthe.
	- Produits pharmaceutiques: Artotec ^®^ (association diclofenac et misoprostol) Aspirine^®^ (Acide acetyl salycilé) Cytotec^®^ (Misoprostol) Sintrom^®^ (anti-vitamine K) Dépakine^®^, (anti-epileptique)
Intramusculaire	- méthergin^®^ (ergometrine) - syntocinon^®^ (ocytocine) - depoprovera^®^ (Medroxyprogesterone acetate)
Trans-vaginale	- Préparations liquides ou tampon à base d'herbes: absinthe, persil, momordique. - Produits chimiques: eau de javel, détergent, permanganate de potassium - Injection intra-utérine du sérum salé hypertonique. - Objets perçants: tiges métalliques, tiges de plantes, sonde urinaire rigide, canule d'aspiration, curette fenêtré.

**Les complications:** Les complications recensées représentent un grand éventail allant d'une simple anémie au décès de la patiente, le Tableau 3 regroupe les complications au fil du temps et leurs pourcentages.

**Tableau 3:** Les complications secondaires à l'avortement non médicalisé

Complications immédiates	Nombre de cas	pourcentage
Rétention trophoblastique	412	91,3%
Anémie aiguë	338	74,9%
Hémorragie	252	55,7%
Plaies cervico-vaginales	70	15,5%
Brulures chimiques	32	7%
Perforations utérines	11	2,4%
Plaies intestinale	1	0,2%
**Complications à moyen terme**		
Endométrite	120	26,6%
Péritonite	45	9,9%
Abcès du Douglas	6	1,3%
Septicémie	4	0,8%
Décès par septicémie	1	0,2%
**Complications à long terme**		
Algies pelviennes	120	26,6%
Dyspareunie	82	18,1%
Synéchies utérines	45	9,9%
imperméabilité tubaires	32	7%
Dépression	24	5,3%
Perdues de vu	158	35%

**Les traitements alloués aux patientes avortées:** La moyenne d'hospitalisation pour les patientes est de trois jours avec des extrêmes allant de un à quinze jours. La révision utérine (Aspiration et/ou curetage) est réalisée chez 412 patientes (91,3%). La réparation des lésions physiques et chimiques vulvo-vaginales et cervicales est effectuée chez 102 patientes (22,5%). 18 malades (3,9%) sont traitées chirurgicalement (laparotomie) pour perforations utérines, plaies intestinale et Abcès du Douglas. 22 cas (4,8%) de transfusions en urgence pour hémorragies et anémies aigue inférieures à 6g d'hémoglobine. Toutes les patientes sont mise sous antibiothérapie et sérum anti tétanique, 124 (27,5%) ont adhéré à la reprise de la vaccination anti-tétanique. 24 femmes (5,3%) ont bénéficié d'un suivie psychiatrique pour dépression post abortum.

## Discussion

Les avortements non médicalisés continuent à progresser dans les pays en voie de développement où l'interruption volontaire de grossesse (IVG) est illégale. Selon l'Organisation mondiale de la santé(OMS) le taux des avortements non médicalisés par rapport au taux général mondial des avortements est passé de 44% en 1995, à 47% en 2003 puis 49% en 2008. C'est la première cause de mortalité maternelle en Ouganda et au Ghana. En Europe où les IVG sont généralement légales il y a une forte disparité entre l'Ouest ou le taux d'avortement n'est que de 12 pour 1000 alors qu'il est de 43 pour 1000 à l'Europe de Est, cette différence s'explique par la faibles utilisation des contraceptives modernes selon le Guttmacher Institute [1–5]. Au Maroc Aucune étude statistique n'existe vraiment en la matière donc la fréquence 3,74% de l'avortement non médicalisé relevée dans notre étude est non représentative de la population générale marocaine. Selon la littérature La tranche d’âge qui a le plus recoure à l'avortement non médicalisé est généralement moins de 20 ans poussée par la peur des parents de la société, la non-reconnaissance de la grossesse par le progéniteur et la législation poussée par les grands courants religieux qui interdisent l'avortement [5–7]. Cela rejoint notre étude ou 70% des patientes ont l’âge entre 16 et 18 ans et les patientes sont conduites vers l'avortement clandestin pour les mêmes raisons décrites dans la littérature en plus du risque d'exclusion du système éducatif qui interdit à toute fille enceinte l'accès aux établissements scolaires. Au-delà de 20 ans la majore partie des avortements non médicalisés sont conséquentes aux problèmes socio-économique associées aux problèmes majeurs, l'interdiction de l'avortement par la loi et le manque de la pratique contraceptive surtout dans les pays africains [2, 8–10]. Les procédés utilisés par les tradipraticiens sont multiples et changent d'un pays à un autre mais ils sont toutes basées sur la genèse des contractions utérine et l'ouverture de l’œuf: les plantes emménagogues (provoquant le flux menstruel) sous formes d'infusion à boire ou des lavements et des tampons en intra vaginal, dans chaque pays on utilise les herbes qui s'y trouvent. Certaines plantes exposent à des intoxications graves pouvant aboutir même au décès; les produits manufacturés: l'eau de Javel, le permanganate de potassium qui donnent des brulures vaginales, les pesticides ou les détergents en liquide qui donnent des intoxications aux organophosphorés; les objets perçants: tige métallique, aiguille à tricoter, tige végétale (branche d'olivier flexible dans notre étude) ou sonde vésicale siliconée; les produits pharmaceutiques: cytotec^®^en Europe, artotec^®^ au Maroc, les antipaludéens en Afrique, une étude en Éthiopie montre que les avortements clandestins sont provoqués dans un tiers des cas par des antibiotiques à doses élevés; Les traumatismes volontaires: coup sur le bas ventre ou saut d'une hauteur élevée [7–10]. Les motifs de consultation sont dominés surtout par le syndrome hémorragique et l'infection, un certain nombre de patientes garde le mutisme sur les conditions de l'avortement, dans notre étude toutes les femmes dévoilent les circonstances de l'avortement mais généralement après l'acte médical de sauvetage (rôle assuré par les assistantes sociales militaires exerçant à l'hôpital) [5–10]. les complications et les séquelles ce sont les mêmes retrouvées dans la littérature que dans notre étude, à cours et à moyen terme les hémorragies, les déchirures du vagin ou du col de l'utérus, la perforation de l'utérus, l'infection voir le décès de la patiente, à long terme la dyspareunie, les douleurs pelviennes chroniques, l'iso-immunisation Rhésus, troubles de fertilité et les problèmes psychologiques.

De nombreuses études scientifiques certifient la nécessité d'une aide psychologique des patientes avortées pour éviter le sentiment de culpabilité, la perte de l'estime de soi, l'insomnie et les troubles sexuels [11–16]. Les avortements non médicalisés génèrent un coût important pour la santé publique à travers la prise en charge de leurs complications et ceci par l'augmentation du taux d'occupation des lits, du budget consacré aux traitements médico-chirurgicaux et la mobilisation du personnel médical et paramédical [2–4, 17]. La législation joue un rôle indirect mais important dans l'augmentation de la fréquence de l'avortement non médicalisé à travers l'interdiction ou la restriction de l'interruption volontaire de la grossesse. L'avortement n'est autorisé pour aucune raison dans 14 pays d'Afrique: (Angola, République centrafricaine, Gabon, Congo Brazzaville, République Démocratique du Congo, Guinée-Bissau, Lesotho, Madagascar, Mauritanie, Somalie, Égypte, Sénégal, Sao Tomé-et-Principe, les iles Maurice). Au Maroc à partir de 2015 la loi autorise l'avortement en cas de malformation fœtale, de viol ou d'inceste, venant s'ajouter à celle déjà permise en cas de danger pour la santé de la mère, mais les avortements en cas de grossesses non désirées de mineures ou grossesse illégitimes sont toujours interdits. En Amérique du nord (USA) certains états essaient même de rendre l'avortement illégal par le biais de référendums, (l'Etat du Dakota a essayée à deux reprises en 2006 et 2008) [17–19]. La majorité des écrits soulève la nécessité de légaliser l'avortement et d'informer l'opinion publique à travers les médias et les programme scolaires (l’éducation sexuelle) du bénéfice de la contraception, ce qui pourrait éviter le recoure aux avortements non médicalisés [20–22].

## Conclusion

L'interdiction de l'interruption volontaire grossesse et l'insuffisance de connaissances des procédés anticonceptionnels par les jeunes filles au Maroc ouvrent largement la voie aux avortements non médicalisées dont les conséquences sont parfois dramatiques comme en témoigne notre étude. La lutte contre ce fléau ne serait possible qu’à travers un dialogue ouvert entre les jeunes et les services socio-sanitaires et éducatifs quant aux moyens de la contraception.

### Etat des connaissance sur le sujet

- Le nombre des avortements non médicalisés observe une augmentation qui est liée au développement démographique dans le monde avec une diminution de la fréquence dans les pays développés, et une nette élévation de la fréquence dans les pays en voie de développement;
- L'engagement du pronostic vital, obstétrical et psychoaffectif est le résultat dramatique de la réalisation non médicalisée des avortements;
- La légalisation de l'avortement et l'amélioration de la qualité des soins ont contribuées en Europe à améliorer la prise en charge des grossesses non désirées.

### Contribution de notre étude a la connaissance

- L'absence de registre des avortements ne permet pas de réaliser des statistiques qui peuvent lever le voile sur la réalité saumâtre des avortements clandestins qui génèrent le décès de plusieurs jeunes femmes;
- L'adolescente non instruite dans un pays ou l'avortement est non légalisé l'expose à s'adresser à des services non compétents causant des préjudices aussi bien physiques que psychosociale;
- La nécessité d'intervenir à plusieurs niveaux pour lutter contre l'avortement non médicalisé en passant par la sensibilisations des jeunes à la contraception, la révision des textes législatifs quant au sujet de l'avortement et l'amélioration de l'accès aux structures sanitaires sans stigmatisation.
